# Pulmonary endarteritis and endocarditis complicated with septic embolism: a case report and review of the literature

**DOI:** 10.1186/s12879-020-4925-z

**Published:** 2020-03-12

**Authors:** Arezoo Khosravi, Zohreh Rostami, Mohammad Javanbakht, Nematollah Jonaidi Jafari, Mohsen Sadeghi Ghahroudi, Mohammad Hassan Kalantar-Motamed, Ramezan Jafari, Behzad Einollahi

**Affiliations:** 1 Atherosclerosis Research Center, Baqiyatallah University of Medical Sciences, Tehran, Iran; 2 Nephrology and Urology Research Center, Baqiyatallah University of Medical Sciences, Tehran, Iran; 3 Health Research Center, Baqiyatallah University of Medical Sciences, Tehran, Iran; 4 Department of Surgery, Baqiyatallah University of Medical Sciences, Tehran, Iran; 5 Department of Radiology, Baqiyatallah University of Medical Sciences, Tehran, Iran; 6 Chemical Injuries Research Center, Baqiyatallah University of Medical Sciences, Tehran, Iran

**Keywords:** Pulmonary endarteritis, Vegetation, Echocardiography, Surgery

## Abstract

**Background:**

Pulmonary endarteritis is a rare clinical phenomenon with congenital heart that can potentially lead to major complications.

**Case presentation:**

We report a 47-year-old man with pulmonary endarteritis. This patient presented with hypertension, chest pain and a previous history of pulmonary valve disease during childhood. Also, eight-months prior, he was hospitalized with dyspnea (Functional Class III), cough, phlegm, and night sweats without fever. Echocardiographic diagnosis in the first transtransthoracic echocardiography (TTE) was intense pulmonary valve stenosis (PVS) an, thus, the pulmonary valve vegetation and PVS, established by transesophageal echocardiography (TEE). He was referred for surgery after 1 weeks of intravenous antibiotic therapy for removal of the vegetation.

**Conclusions:**

Finally he was asymptomatic at 3-months of follow-up and was clinically in good condition. Therefore, the detection of infective endocarditis of the lung valve must not lengthy be prolonged.

## Background

Pulmonary endarteritis is an uncommon incident even in patients with congenital heart that can potentially lead to major complications, and its frequency has declined dramatically over the last thirty years [1, 2]. Furthermore, septic pulmonary embolism as a complication of infective endocarditis is an infrequent condition and a rare disorder that commonly presents with symptoms including fever, cough, and hemoptysis, chest pain, purulent sputum, and dyspnea [3, 4]. This disease were usually diagnosed to be related to infected heart valves, septic intravenous drug, thrombophlebitis,, osteomyelitis, pulmonary artery catheter or infected pacemaker wires, bacterial endocarditis, and Staphylococcus species [2, 3, 5]. In addition, the coexistence of pulmonary endarteritis and endocarditis with septic embolism is a disorder very rare. Therefore, we present here a man with disorders mentioned above due to extremely scarce disorders.

## Case presentation

A 47-year-old man was referred to the cardiology department with a history of hypertension and chest pain for further evaluation of his condition. Eight months ago, he suffered from dyspnea (Functional Class III), cough and phlegm, night sweats without fever. In addition, during the past two months, exacerbation of the edema symptoms (grade III), there was also positive history of anorexia, severe weakness and fatigue, weight loss and chest pain. Moreover, the patient had a previous history of pulmonary valve disease of childhood. Furthermore, he had no known risk factors except for congenital valvular heart disease, which was ignored from childhood. Plus, there was no history of smoking, narcotic medication, and alcohol. He was under treatment with amlodipine, losartan, spironolactone, carvedilol, furosemide, atorvastatin, and aspirin. On physical examination, heart rate 85 beats/min, respiratory rate 20 beats/min and his blood pressure was 130/80 mmHg. On inspection, the jugular vein pressure and lung were normal. Furthermore, cardiac auscultation demonstrated a continuous systolic murmur (grade 4/6) was best heard in the second left intercostal space. Plus, there were no peripheral stigmata of infective endocarditis, but on abdominal examination, there were splenomegaly and hepatomegaly.

The echocardiography revealed the normal sinus rhythm, normal axis and incomplete right bundle branch block. Laboratory investigations indicated WBC = 10,100 micL,POLY = 77%,Hemoglobin = 10.1 g/dl,MCV = 80 ft.,Platelet = 207,000/mm^3^,CRP = 77 mg/L, ESR = 90 mm/h,Urine analysis normal, Renal function test normal,FBS = 90 mg/dl, TG = 86 mg/dl,Cholesterol = 108 mg/dl, LDL = 60 mg/dl,HDL = 27 mg/dl, AST = 43 U/L,ALT = 28 U/L.

Blood culture yielded streptococcus sp. sensitive to ampicillin, ceftriaxone, and vancomycin. All results for HBS Antigen, HCV Antibody, HIV antibody, PPD, Wright, 2 mercaptomethanol reductions were negative. Also serum complement concentration, ANA (anti-nuclear antibody), Anti-double strand DNA were in normal limit.

Chest X ray findings showed increased bronchovascular markings. In Color Doppler ultrasound of the lower extremities, there was no deep vein thrombosis (DVT)

### Echocardiography findings

#### Transthoracic echocardiography (TTE) + Tissue Doppler imaging (TDI)

1. Left ventricle (LV): LV size is moderate enlarged with normal systolic function. There is mild left ventricular hypertrophy (LVH)
2. Right ventricle (RV): RV size is moderate enlarged with mild to moderate systolic dysfunction. There is severe RVH. D-shaped septum consistent with pressure overload.
3. Left atrium (LA): LA size is normal.
4. Right atrium (RA): RA size is mild to moderate enlarged.
5. Mitral valve (MV): MV is normal with no mitral regurgitation (MR). There is no mitral stenosis (MS).
6. Aortic valve (AV): AV is thickened and calcified with no aortic insufficiency (AI) there is no aortic stenosis (AS).
7. Tricuspid valve (TV): TV is normal with moderate tricuspid regurgitation (TR). There is no tricuspid stenosis (TS).
8. Pulmonary valve (PV): PV is thick and dome shaped with no pulmonary insufficiency (PI). There is severe pulmonary valve stenosis (PVS).
9. Inferior vena cava (IVC): IVC size is normal and more than 50% respiratory collapse.
10. Pericardium: Pericardium is normal with trivial pericardial effusion.

Echocardiographic diagnosis in the first TTE was severe PS (PPG = 110 mmHg).

#### Transesophageal echocardiography (TEE) + contrast

1. LV: LV size is moderate enlarged with normal systolic function. There is mild LVH
2. RV: RV size is moderate enlarged with mild to moderate systolic dysfunction. There is severe right ventricular hypertrophy (RVH). D-shaped septum consistent with pressure overload.
3. LA: LA size is mild to moderate enlarged.
4. RA: RA size is moderate enlarged.
5. MV:MV is thickened with no MR. there is no MS.
6. AV: AV is thickened and calcified with no AI there is no AS.
7. TV: TV is thickened with moderate TR. There are no TS.
8. PV: PV is thick and dome shaped with mild to moderate PI. There is severe PS.
9. Interventricular septum (IVS): IVS has no VSD
10. Interatrial septum (IAS): IAS is thickened there is small size PFO, by contrast study.
11. IVC: IVC size is normal and more than 50% respiratory collapse.
12. Pericardium: Pericardium is normal with trivial pericardial effusion.
13. Left arterial appendage (LAA): LAA is normal with no thrombus. LAA velocity: 94.8 cmls
14. Aorta: descending, abdominal aorta and aortic arch have normal size and flow.

**Fig. 1:**
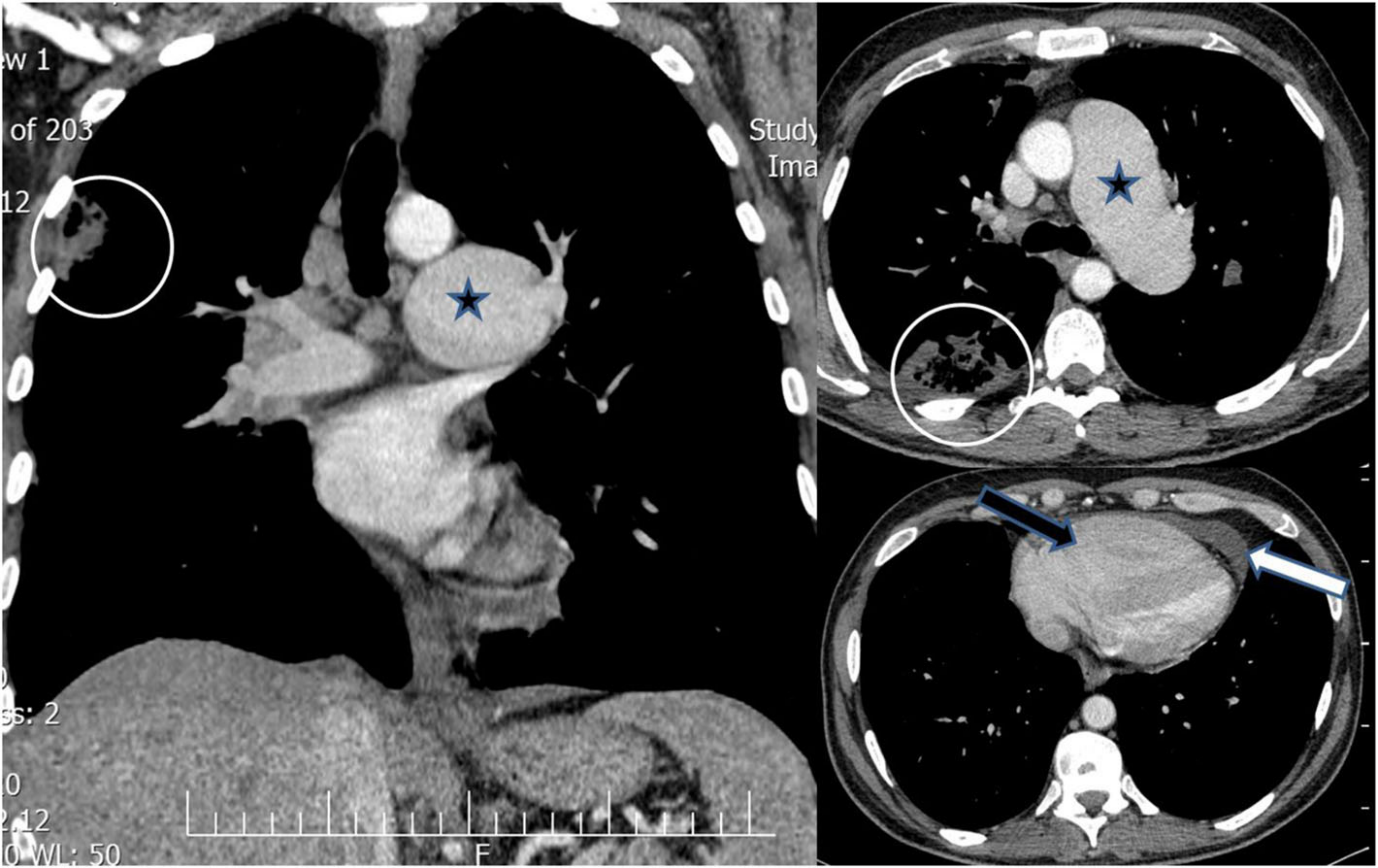
Axial and reconstructed coronal contrast-enhanced chest CT scan (mediastinal window): severe enlargement of main pulmonary artery and left pulmonary artery (black star) compatible post stenotic dilatation due to severe pulmonary stenosis(PS), large cavitary lesion (white circle) at RUL in favor of septic emboli,pericardial effusion (white arrow) and right ventricular enlargement (black arrow)

Diagnosis with TEE was severe PS(PPG = 110) with large mobile mass(1.5*1.8 cm) on arterial side of PV and another very large mobile mass (2.5*0.9 cm) was attached to the luminal of main of PA at the distal part (5 cm above pulmonic valve) at jet site of PS (Fig. 1).

**Fig. 2:**
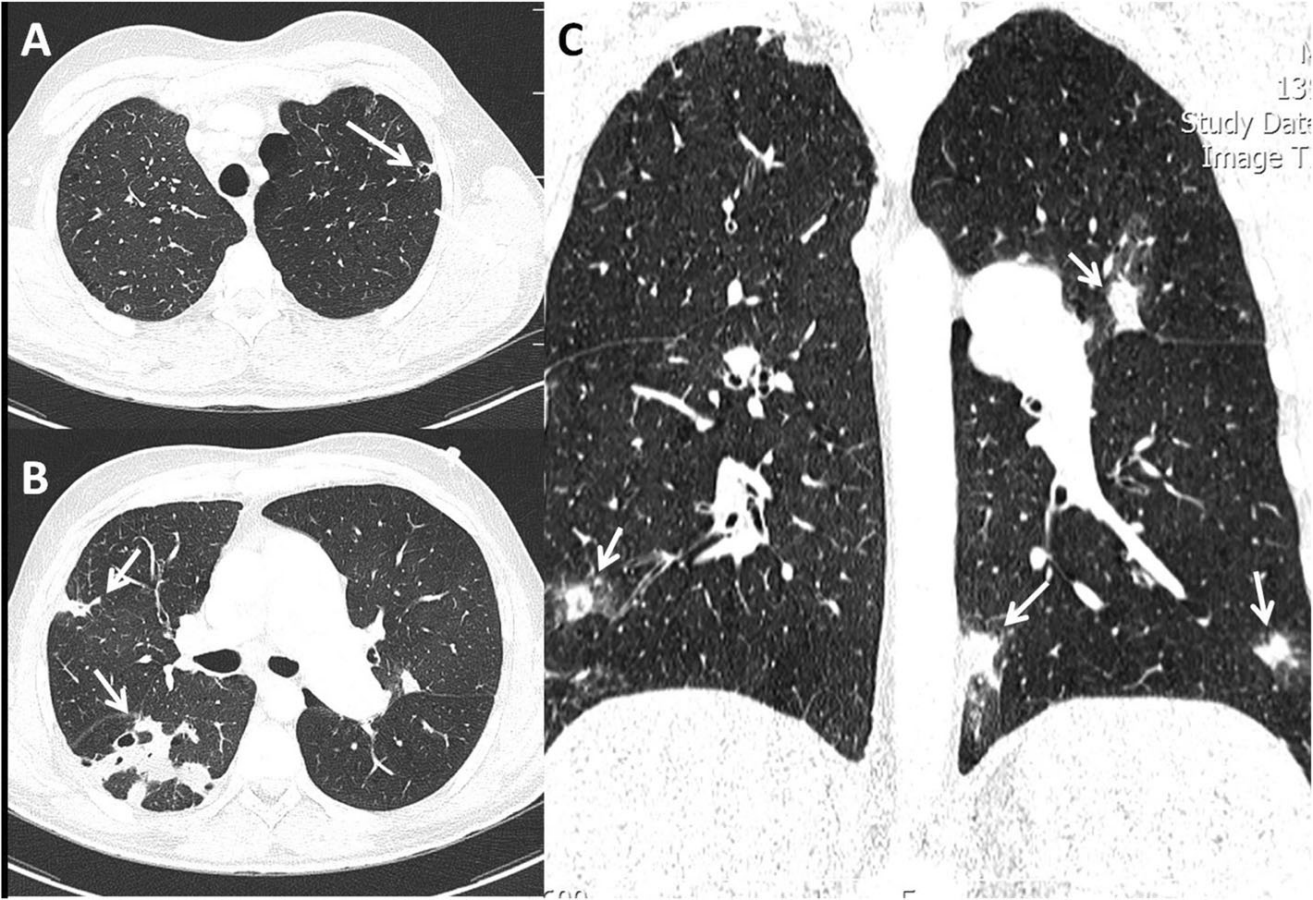
**a-c** axial and reconstructed coronal chest CT scan (lung window): multiple ill-defined pulmonary nodules with peripheral ground-glass halo and cavitary changes at peripheral zone of both lungs (white arrows) compatible with septic emboli

**Fig. 3:**
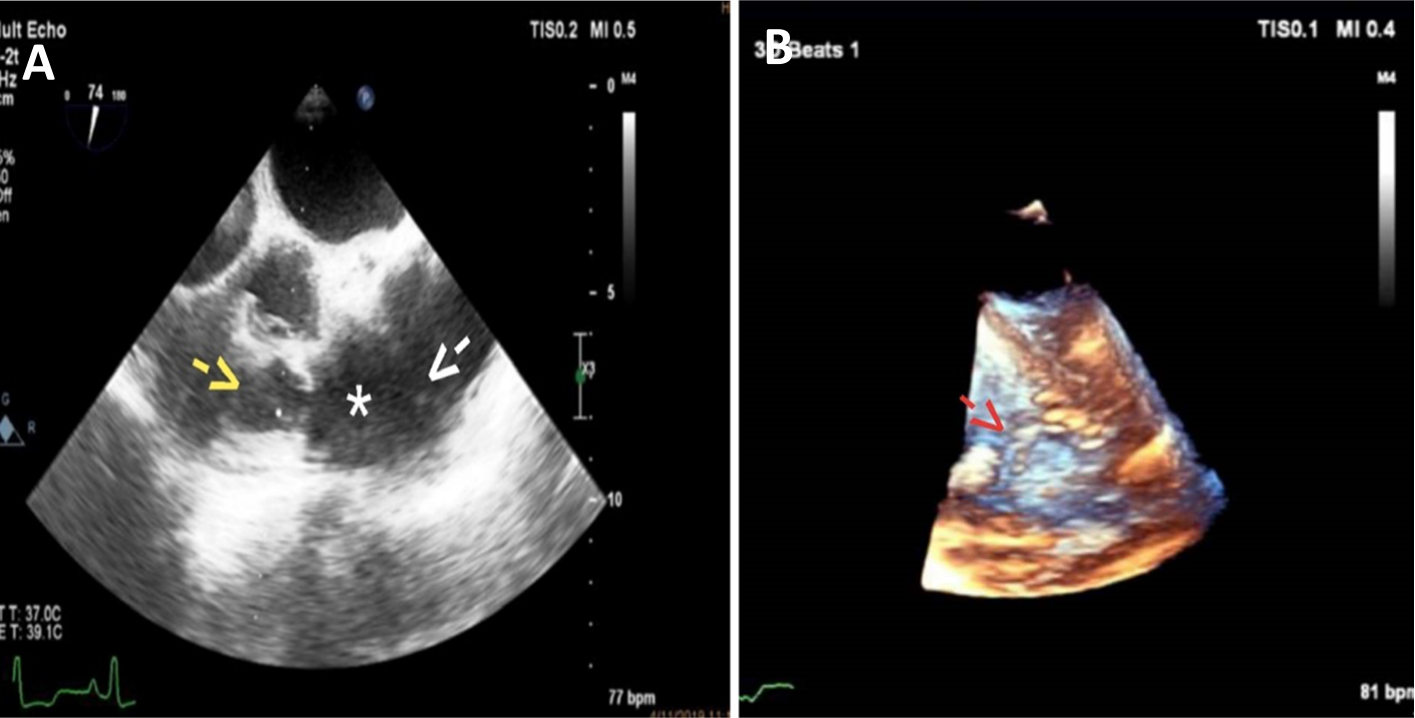
**A**- 2D Transesophageal echocardiography (TEE) of dome shaped pulmonic valve with vegetation (Yellow arrow) and post stenotic dilatation of pulmonary artery (*) and vegetation on pulmonary artery wall (Endarteritis) white arrow, 74 degree. **B** -3D TEE echoardiography of vegetation in main pulmonary artery wall (Red arrow)

The diagnosis of pulmonary endarteritis and endocarditis was made according to the lung CT scan and pulmonary and coronary artery CT angiography findings (Figs. 2 and 3). Antibiotic therapy was continued for 1 week in hospital, and the patient was referred for pulmonary valve replacement with complete resection of large vegetations. We empirically started vancomycin 1 g q 12 h and meropenem 1 g q 8 h due to extracardiac complication (pulmonary septic embolism) and after 48 h when antibiogram results was reported we didn’t change antibiotic therapy. During 2 weeks after surgical management and antibiotic therapy, blood culture (2 blood sample with 1 h interval) repeated every 4 days until negative blood culture obtained(Totally 4 times blood culture was positive).

Surgery was performed and a large 3*2 cm highly mobile strand like vegetation was seen adherent to the arterial wall about 5 cm above PV annulus. This was completely removed from the arterial wall with no residue and sent for culture and pathologic inspection. Then the pulmonary valve was inspected and found to be severely calcified and with multiple vegetations adherent to the deformed and stenotic leaflets, so the valve was resected and sent for culture and pathologic inspection. Then pulmonary valve was replaced with a #23 On-X valve. Then the right ventricular outflow tract (RVOT) was repaired.After negative blood culture, antibiotic therapy lasted for 6 weeks. He discharged after 3 weeks with a good condition while antibiotic therapy continued outpatient. In addition,the clinical course of pulmonary septic emboli after surgery,this was that in the first week of treatment he became afebrile, the patient’s pulmonary symptoms recovered gradually, leukocytosis and ESR decreased and chest X ray resolved slowly over 6–8 weeks.

ESR(40 mm/h) and CRP (37 mg/L)returned to normal values after the operation. He was discharged on 21th post-operative day with better echocardiography findings (Acceptable gradient of mechanical prosthesis PV (PVMG: 14 mmHg, PVPG: 20 mmHg), mild turbulence of rRVOT), normal diastolic function) and was asymptomatic at 3-months of follow-up and was clinically in good condition.

## Discussion and conclusions

Pulmonic valve endocarditis is a rare clinical phenomenon [1, 2]. The principal predisposing factors for pulmonic valve infective endocarditis has been recognized in adult patients underwent the intravenous medication, central venous catheters, alcoholism, chronic hemodialysis, hepatic transplantation, celiac disease, and systemic infection with bacterial agents [2, 5–7]. Moreover, according to the study of Georges et al., 2018 revealed that there are many prognostic factors for endocarditis such as:age (e.g,  > 45 years),cause of ICU admission (e.g. septic shock, cardiac, respiratory and renal failure and etc.), source of infection (e.g. cutaneous and etc.),and pathogen agents (e.g. Fungal, staphylococcus and streptococcus and etc.) [8]. A study by Cuenza et al., 2014 suggested that clinical examinations, bacteremia confirmed by blood culture, and echocardiographic imaging are important in the detection of pulmonary endarteritis, they also reported that severity and the degree of pulmonary valve stenosis can be diagnosed by Color Doppler imaging, and also, in this report indicates that rigorous echocardiographic features can demonstrate the presence of vegetation located at the pulmonary artery [7]. In agreement with findings above, transthoracic and esophageal echocardiography evidence of our patient presented with severe pulmonary stenosis related to pulmonary endarteritis together with vegetation adherent to pulmonary valve confirmed by the lung CT scan and pulmonary and coronary artery CT angiography imaging.

In patients with infective endarteritis need the administration of antibiotics usually intravenously for 4–8 weeks after the bacterial identification. However, long-term antibiotic therapy may cause renal failure and other adverse effects [9]. In present case, his blood cultures exhibited the presence of Streptococci SP., so surgical management has been considered due to the uncontrollable infection. Also, surgical approach should be considered in patients with progressive heart failure or bacterial infection that is resistant to antibiotics or reveals vegetation greater than 10 mm.

Importantly, primary clinical distrust and a precise echocardiography imaging study enabled us establish the diagnosis and made us deployed effective therapy and promoted a good outcome. In adult patients, vegetations in the pulmonary valve is not clearly represented by TTE. TEE seems to be more sensitive and the best diagnostic tool for vegetation detection [1, 10, 11].

In summary, this report illustrates that in whole patients the lung valve should be correctly evaluated by TTE and TEE with clinical suspicion of pulmonary endarteritis, particularly in the attendance of extended signs associated with infectious processes such as Streptococcus sp., exclusively if they have predisposing heart disease. The detection of infective endocarditis of the lung valve must not lengthy be prolonged Therefore, this case highlights the significance of recognizing the possibility of these complications. Physicians should be knowledgeable and alert in their diagnosis using clinical and echocardiogram techniques, along with rapid antibiotic treatment, and the provision of surgical options for management.

## Data Availability

All data generated or analyzed during this study are included in this published article.

## Abbreviations

AI: Aortic insufficiency
AS: Aortic stenosis
AV: qAortic valve
IAS: Interatrial septum
IVC: Inferior vena cava
IVS: Interventricular septum
LA: Left atrium
LAA: Left arterial appendage
LV: Left ventricle
LVH: Left ventricular hypertrophy
MR: Mitral regurgitation
MS: Mitral stenosis
MV: Mitral valve
PI: Pulmonary insufficiency
PV: Pulmonary valve
PVS: Pulmonary valve stenosis
RA: Right atrium
RV: Right ventricle
RVH: Right ventricular hypertrophy
RVOT: Right ventricular outflow tract
TDI: Tissue Doppler imaging
TEE: Transesophageal echocardiography
TR: Tricuspid regurgitation
TS: Tricuspid stenosis
TTE: Transtransthoracic echocardiography
TV: Tricuspid valve
